# scNetViz: from single cells to networks using Cytoscape

**DOI:** 10.12688/f1000research.52460.1

**Published:** 2021-06-07

**Authors:** Krishna Choudhary, Elaine C. Meng, J. Javier Diaz-Mejia, Gary D. Bader, Alexander R. Pico, John H. Morris

**Affiliations:** 1 Institute of Data Science and Biotechnology, Gladstone Institutes, San Francisco, California, 94158, USA; 2 Department of Pharmaceutical Chemistry, University of California San Francisco, San Francisco, California, 94143, USA; 3 Princess Margaret Cancer Centre, University Health Network, Toronto, Ontario, M5G 2M9, Canada; 4 The Donnelly Centre, University of Toronto, Toronto, Ontario, M5S 3E1, Canada; 5 Phenomic AI, Toronto, Ontario, M5G 0B7, Canada; 6 Department of Molecular Genetics, University of Toronto, Toronto, Ontario, M5G 1A8, Canada; 7 Department of Computer Science, University of Toronto, Toronto, Ontario, M5T 3A1, Canada

**Keywords:** scRNA-seq, Single cell, Expression analysis, Cytoscape, App, Network biology

## Abstract

Single-cell RNA-sequencing (scRNA-seq) has revolutionized molecular biology and medicine by enabling high-throughput studies of cellular heterogeneity in diverse tissues. Applying network biology approaches to scRNA-seq data can provide useful insights into genes driving heterogeneous cell-type compositions of tissues. Here, we present *scNetViz* ***— *** a Cytoscape app to aid biological interpretation of cell clusters in scRNA-seq data using network analysis. *scNetViz* calculates the differential expression of each gene across clusters and then creates a cluster-specific gene functional interaction network between the significantly differentially expressed genes for further analysis, such as pathway enrichment analysis. To automate a complete data analysis workflow, *scNetViz* integrates parts of the *Scanpy* software, which is a popular Python package for scRNA-seq data analysis, with Cytoscape apps such as *stringApp*, *cyPlot*, and *enhancedGraphics*. We describe our implementation of methods for accessing data from public single cell atlas projects, differential expression analysis, visualization, and automation. *scNetViz* enables users to analyze data from public atlases or their own experiments, which we illustrate with two use cases. Analysis can be performed via the Cytoscape GUI or CyREST programming interface using R (RCy3) or Python (py4cytoscape).

## Introduction

Single-cell RNA sequencing (scRNA-seq) has yielded significant insights into mechanisms regulating diverse biological systems. ^1^ This technology captures transcriptome-wide expression profiles of single cells, which can be used to cluster cells and identify the biological features that distinguish the clusters. ^2^ With continued technological advances and cost efficiencies, scRNA-seq is becoming increasingly common and new data are being generated at a rapid pace. Global efforts such as the Human Cell Atlas, ^3^ which aims to map a healthy human, and the EMBL-EBI Single Cell Expression Atlas, ^4^ which organizes published datasets across multiple species and provides access to results from standardized analyses, are growing rapidly. To study cell types in scRNA-seq data, it is useful to compare cell clusters to identify cell-type-specific gene expression markers and genes associated with a phenotype. ^2, 5^ Such analysis typically provides a ranking of genes based on the statistical significance of differential expression. Besides this, applying network biology approaches can yield helpful insights for biomedical applications. ^6, 7, 8, 9^ In this direction, some methods enable inference of cell-type-specific regulatory networks from the experimental data. ^10, 11, 12, 13^ In contrast, another approach is to filter gene networks from independent experiments or public databases using single-cell data for cell-type-specific insights. ^7, 14^

Public databases such as the STRING database ^15^ contain high-quality and well-organized information about published interaction networks. Here, we describe *scNetViz*— an interactive desktop Java application that harnesses the extensive network visualization and analysis capabilities of Cytoscape for single-cell data analysis. ^16, 17^ *scNetViz* takes a network filtering approach as mentioned above. It enables users to load their own scRNA-seq data or pull such data from public atlases, cluster cells, run differential gene expression analysis, perform dimensionality reduction of expression matrices for data visualization, generate heatmaps and violin plots of gene expression data, and examine the associated gene interaction networks from the STRING database. Below, we describe the methods implemented in *scNetViz*, provide two use cases to illustrate the user interface and programmatic access using R and Python, and discuss plans for future development. *scNetViz* enables scientists who may not be experts in scripting to explore the data and to develop biological hypotheses. Further, it provides a convenient interface for integrating gene network information with scRNA-seq datasets, which can save time for researchers.

## Methods

### Overview

*scNetViz* consists of two main components, a Java-based Cytoscape app and a web service implemented on a compute cluster at the Resource for Biocomputing, Visualization and Informatics (RBVI) at the University of California, San Francisco. In addition, the app portion uses several other Cytoscape apps, including *stringApp*, ^18^ *cyPlot*, ^19^ and *enhancedGraphics*. ^20^

The Cytoscape app provides the following functionality:

1. Acts as the graphical front-end for *scNetViz*, including providing various dialogs for interactive users and commands for automation uses.
2. Manages all of the data that has been imported. This includes acting as a client for the Single Cell Expression Atlas ^4^ and Human Cell Atlas ^3^ data portal REST services as well as providing users the option to import single cell expression matrices and category data (e.g. tissue, cluster information, experimental condition, etc.) from local files.
3. Calculates the differential gene expression for a chosen category (e.g. a specific clustering resolution or sample characteristic such as disease vs. non disease): a separate calculation for each group of cells (e.g. cluster) within that category vs. the set of all other cells (e.g. clusters) within that same category.
4. Fetches functional association networks from STRING for the protein products of significantly differentially expressed genes.
5. Interfaces with the RBVI web service described above to calculate cell plots based on UMAP ^21^ or t-SNE. ^22^
6. Provides a Java-based implementation of t-SNE for smaller experiments (can be faster than the web service).
7. Interfaces with *cyPlot*, a Cytoscape app that displays various plots in the Cytoscape web browser, to visualize all UMAP and t-SNE embeddings as well as volcano plots and heatmaps.
8. Finally, it also interfaces with the RBVI web service to calculate clusters based on either the Louvain or Leiden algorithms as implemented in *Scanpy*. ^23^

The RBVI web service component provides a simple wrapper around *Scanpy* that exposes REST endpoints for:

1. Calculating a UMAP embedding of an scRNA-seq matrix,
2. Calculating a tSNE embedding of an scRNA-seq matrix,
3. Calculating a Leiden clustering of an scRNA-seq matrix,
4. Calculating a Louvain clustering of an scRNA-seq matrix.

Matrices are passed as compressed MatrixMarket files ^24^ and parameters may be provided to simplify and normalize the matrix in a pre-processing step, in addition to parameters related to the appropriate algorithm.

### Cytoscape app

The Cytoscape App provides the main front-end, data management, and orchestration for *scNetViz.* Importantly, the app also exports Cytoscape commands so that *scNetViz* can be used by CyREST ^25^ packages such as RCy3 ^26^ and py4cytoscape. ^27^ Below, we discuss five of the app functions: data access, differential gene expression calculation, plot generation, network creation, and automation via R and Python. The various user interface components are all implemented in Java Swing, use the Cytoscape Java Desktop API, and are shown in the use case descriptions.

### Data access

*scNetViz* supports three types of data: cell by gene matrices (typically read in MatrixMarket format), experiment-level metadata including organism, number of cells in the experiment, and category-level metadata such as cell cluster assignments, disease state, cell type, tissue, ethnicity, which is typically in comma-separated value (CSV) format. *scNetViz* has a **sources** module, which includes submodules for each supported data source. A source module provides the tasks necessary to import data for an experiment or category, enumerates the available experiments and displays them. We have currently implemented support for three sources: EMBL-EBI’s Single Cell Expression Atlas, the Human Cell Atlas data portal, and local files. The former two are public sources and are referred to as *GXA* and *HCA*, respectively, in *scNetViz* commands. They provide support for browsing experiments, while the local source provides methods to read category and experiment files. Since different category data might be in a different orientation (typically clusterings files are organized with the clusters in rows and cells in columns, but cell metadata and demographics are organized with the cells in rows and categorical information in the columns), we provided the ability to read in the CSV and transpose the resulting matrix, which may be needed to feed the data to downstream analysis steps.

To import data from the two public repositories, we rely on their published REST interfaces. The Single Cell Expression Atlas provided the simplest interface as specific endpoints for the matrices, clusters, and experimental-design:

• To enumerate all experiments:
https://www.ebi.ac.uk/gxa/sc/json/experiments. This returns a JSON file that contains an entry for each experiment and significant metadata about the experiment.
• To import an experiment:
 https://www.ebi.ac.uk/gxa/sc/experiment/\%s/download/zip?fileType=normalised (where ”%s” is the accession identifier for the experiment). This returns a zip archive with the matrix, cell barcodes, and gene identifiers.
• To import the clusters provided:
https://www.ebi.ac.uk/gxa/sc/experiment/\%s/download?fileType=cluster. This returns a CSV file of clustering results for various resolutions with an indication of the best resolution according to GXA.
• To import the experimental design:
https://www.ebi.ac.uk/gxa/sc/experiment/\%s/download?fileType=experiment-design. This returns a CSV file that is transposed before being displayed.

Data access from the Human Cell Atlas recently transitioned to a new data coordination platform (DCP) API. *scNetViz* currently uses the ”DCP1” API, and we will transition to ”DCP2” in the near future. To access the DCP1 data:

• We can enumerate all experiments using the URL: https://service.azul.data.humancellatlas.org/index/projects?catalog=dcp1 but, unlike the Single Cell Expression Atlas, the Human Cell Atlas contains raw data (e.g. Fastq and BAM files) as well as calculated matrices. To restrict our enumeration to include only matrix data, we need to include a filter, which is encoded as part of the URL. We use the following filter.

{"fileFormat":{"is":["matrix"]}}

  It is encoded in the URL as using the ”filters” query option: https://service.azul.data.humancellatlas.org/index/projects?catalog=dcp1\&filters={"fileFormat":{"is":["matrix"]}}. This query results in a JSON file that includes metadata about each experiment and a link to the expression matrix file itself. Because in the Human Cell Atlas a single ”Project” may include multiple matrices, one for each tissue or organ that was investigated, we split those projects into independent experiments.
• To load the experiment data, we follow the URL provided by the experiment-level metadata. The result is a zip file that contains the matrix itself in MatrixMarket format, the gene identifiers, and a file that contains both the cell barcodes and categorical data for each cell.

### Differential expression calculation

As shown below, the key function of *scNetViz* is to calculate the differential expression of each gene across categories and then use the highly differentially expressed genes (possible marker genes) to construct a gene functional interaction network. We calculate differential gene expression using Mann-Whitney U test and represent it as the log _2_(fold change) where fold change is the ratio of the mean expression of a gene in a single group within a category vs. the mean expression of that gene in the comparison set of all other groups within that same category. For example, if the chosen category is a clustering with K=5 classes, then each of the five classes is one group within the category. We provide two cutoff values to reduce noise in the resulting gene interaction network. First, a gene must be expressed in at least 10% of the cells in the group or 10% of the cells in the set of all other groups in the category. Second, we have a minimum log _2_(fold change) cutoff that drops any changes that are beneath that threshold. All of this data is provided in a table that is created and provided to the user.

### Plot generation

All of the plots provided by *scNetViz* are generated by the *cyPlot* Cytoscape App. ^19^ *cyPlot* uses the built-in Cytoscape web browser and the plotly.js Javascript library to display plots. All of the data is passed to *cyPlot* using its automation interface. For example, below is the *scNetViz* method to create a violin plot.


 public static void createViolinPlot(ScNVManager manager,
                     String names, String data,
                     String groups, String title,
                     String xlabel, String ylabel,
                     String accession,
                     boolean showAll, double jitter) {
 Map<String, Object> argMap = new HashMap<>();
 argMap.put("data", data);
 argMap.put("editor", "false");
 argMap.put("xLabel",xlabel);
 argMap.put("yLabel",ylabel);
 argMap.put("title",title);
 argMap.put("names",names);
 argMap.put("groups",groups);
 argMap.put("id",accession);
 if (showAll)
   argMap.put("showAll","true");
 else
   argMap.put("showAll","false");
 argMap.put("jitter",jitter);
 argMap.put("selectionString",
        "scnetviz select accession=\""+accession+"\" genes=%s");
 manager.executeCommand("cyplot", "violin", argMap);
}


All of the arguments in the above code chunk are JSON strings that are consumed by *cyPlot* and passed on to the plotly.js library for display.

### Network creation

Once the calculation of differential expression is complete, *scNetViz* can create networks to show the functional interactions among the proteins encoded by the differentially expressed genes. This proceeds in two steps. First, the STRING network of a user-specified number (default 200) of top differentially expressed genes for each group within the category is created by using the Cytoscape automation interface of the *stringApp.* The method that does this is:


 private void createStringNetwork(Object cat, String name,
                   List<String> geneList,
                    Map<Object, List<String>> geneMap,
                   TaskMonitor monitor) {
 monitor.setTitle("Retrieving STRING network for: "+name);
 monitor.showMessage(TaskMonitor.Level.INFO,
             "Retrieving STRING network for: "+name);
 Map<String, Object> args = new HashMap<>();
 args.put("query", listToString(geneList, ""));
 args.put("species",
    diffExp.getCurrentCategory().getExperiment().getSpecies());
 args.put("limit", "0");
 args.put("includesViruses", "false");
 manager.executeCommand("string", "protein query", args,
       new RenameNetwork(diffExp, cat, name,
                 geneList, geneMap, false, monitor),
               true);
 cyEventHelper.flushPayloadEvents();
}


This creates the argument map and then calls the executeCommand method, which is a wrapper method that we implemented around the Cytoscape org.cytoscape.command.CommandExecutorTaskFactory. The *stringApp* queries STRING and creates the network. The second part of the process extracts significantly differentially expressed genes in a single group from the network that contains the top differentially expressed genes in a category. This is done using the Cytoscape API to create subnetworks and provide styles that color nodes based on their differential expression in a given group.

### Automation

Similar to the automation functionality that *scNetViz* uses, described above, *scNetViz* provides its own automation commands, useful for scripts to control *scNetViz* operations. The following is a list of available commands (details available in the Swagger documentation (Help → Automation → CyREST Command API)).

**List of scNetViz Commands**

help scnetviz
Available commands:

scnetviz add file category     * Add a category to an experiment from a file*
scnetviz add leiden category    *Calculate the Leiden clustering*
scnetviz add louvain category   *Calculate the Louvain clustering*
scnetviz calculate diffexp      *Calculate the table of differential gene expression*
scnetviz calculate draw_graph   *Calculate the graph layout embedding*
scnetviz calculate tsne         *Calculate the t-SNE embedding (remote)*
scnetviz calculate tSNE         *Calculate the tSNE embedding (local)*
scnetviz calculate UMAP         *Calculate the UMAP embedding (remote)*
scnetviz create all             *Calculate differential expression and create networks*
scnetviz create network         *Create the networks for differentially expressed genes*
scnetviz delete experiment      *Remove an experiment*
scnetviz export category        *Export a currently loaded category table*
scnetviz export diffexp         *Export a differential expression table*
scnetviz export experiment      *Export a currently loaded experiment table*
scnetviz list experiments       *List the currently loaded experiments*
scnetviz list gxa entries       *List all Single Cell Expression Atlas (GXA) entries available*
scnetviz list hca entries       *List all Human Cell Atlas (HCA) entries available*
scnetviz load experiment file   *Load an experiment from a file*
scnetviz load gxa experiment    *Load an experiment from the EBI Single Cell Expression Atlas*
scnetviz select                 *Select genes or assays in current tables*
scnetviz show cell plot         *Display the cell plot (UMAP, t-SNE) for a single experiment*
scnetviz show diff plot         *Display the differential expression plot for a single experiment*
scnetviz show experiment        *Show a currently loaded experiment*
scnetviz show experiment table  *Display the experiment table for a single experiment*

As an example workflow, one might execute the following commands:


# Load experiment "E-CURD-3" from the EBI Single Cell Expression Atlas
scnetviz load gxa experiment accession="E-CURD-3"

# Calculate the differential expression using the default values:
#   category=Cluster
#   categoryRow=4 (or whatever row has "True")
#   logGERCutoff=0.5
#   minPctCutoff=10
scnetviz calculate diffexp accession="E-CURD-3"

# Create the networks using the default values:
#   fdrCutoff=0.5
#   maxgenes=200
#   positiveOnly=false
#   log2FCCutoff=1.0
scnetviz create network accession="E-CURD-3"


### Web service

As discussed above, the web service component is primarily a wrapper around *Scanpy* that exposes a limited set of the *Scanpy* functionality appropriate to the needs of *scNetViz.* In each case, the web service collects the data and arguments, calls *Scanpy* pre-processing routines as appropriate, and then calls the *Scanpy* implementation of the algorithm. By default, the matrix will be normalized, log-transformed, restricted to only the most variable genes, and scaled. The default minimum of 100 genes/cell and a minimum of at least 1 cell for each gene are also used. The parameters to control pre-processing may be changed by opening up the **Advanced pre-processing parameters** section of the UMAP, t-SNE, Louvain, or Leiden dialogs, which enables the user to change any of the pre-processing defaults.

The web service implements the algorithms using the appropriate scanpy.tl methods (scanpy.tl.tsne, scanpy.tl.umap, scapy.tl.leiden, and scanpy.tl.louvain). In all cases, the matrix is pre-processed and passed to the appropriate *Scanpy* module. We expose the most useful parameters in each case. For UMAP, we expose the number of neighbors and the minimum distance parameters; for t-SNE, we expose the perplexity, the number of initial dimensions, the early exaggeration parameter and the learning rate. Note that if the number of initial dimensions is set to -1, a PCA will be calculated first to reduce the dimensionality of the matrix, which can improve the resulting t-SNE embedding. For Leiden and Louvain, we expose the number of neighbors to use to calculate the neighborhood graph before clustering (see scanpy.pp.neighbors). See the *Scanpy* documentation for a more detailed explanation of the effects of these routines and parameters.

### Use Cases

We have prioritized providing access to the Single Cell Expression Atlas and Human Cell Atlas. ^3, 4^ Additionally, researchers can import normalized and clustered scRNA-seq data from local files. The following workflows demonstrate two use cases using either the Single Cell Expression Atlas or local files, and illustrate the utility of *scNetViz* in integrating network information with scRNA-seq data. Scripted versions of these workflows are also available as R Markdown documents and Jupyter notebooks, which leverage the RCy3 ^26^ and py4cytoscape ^27^ packages, respectively. They require Cytoscape (3.7.1 or later), CyREST (3.8.0 or later), and R (version 4.0 or later) or Python (version 3.6 or later). User documentation is available at: https://www.cgl.ucsf.edu/cytoscape/scNetViz/index.shtml. Downloadable notebooks of these workflows are available in R and Python at http://automation.cytoscape.org.

### Use Case #1: Loading data from the EMBL-EBI Single Cell Expression Atlas for scNetViz analysis

In this example, we will browse the Single Cell Expression Atlas from within Cytoscape, explore a particular dataset, perform differential expression analysis based on one of the provided cell annotation categories, generate networks from the top differentially expressed genes for each group within the chosen category, and functionally characterize and visualize the networks.

**Figure 1.**
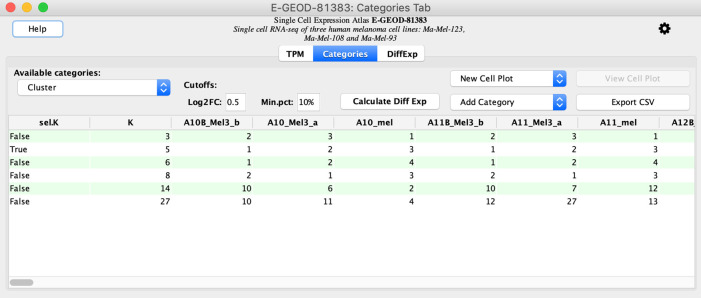
The Categories tab shows the grouping of cells for differential analysis and visualization purposes. The menu labeled **New Cell Plot** contains options for generating UMAP and t-SNE plots. Note that these results are for data from the Single Cell Expression Atlas release 15 (March, 2021).

**Figure 2.**
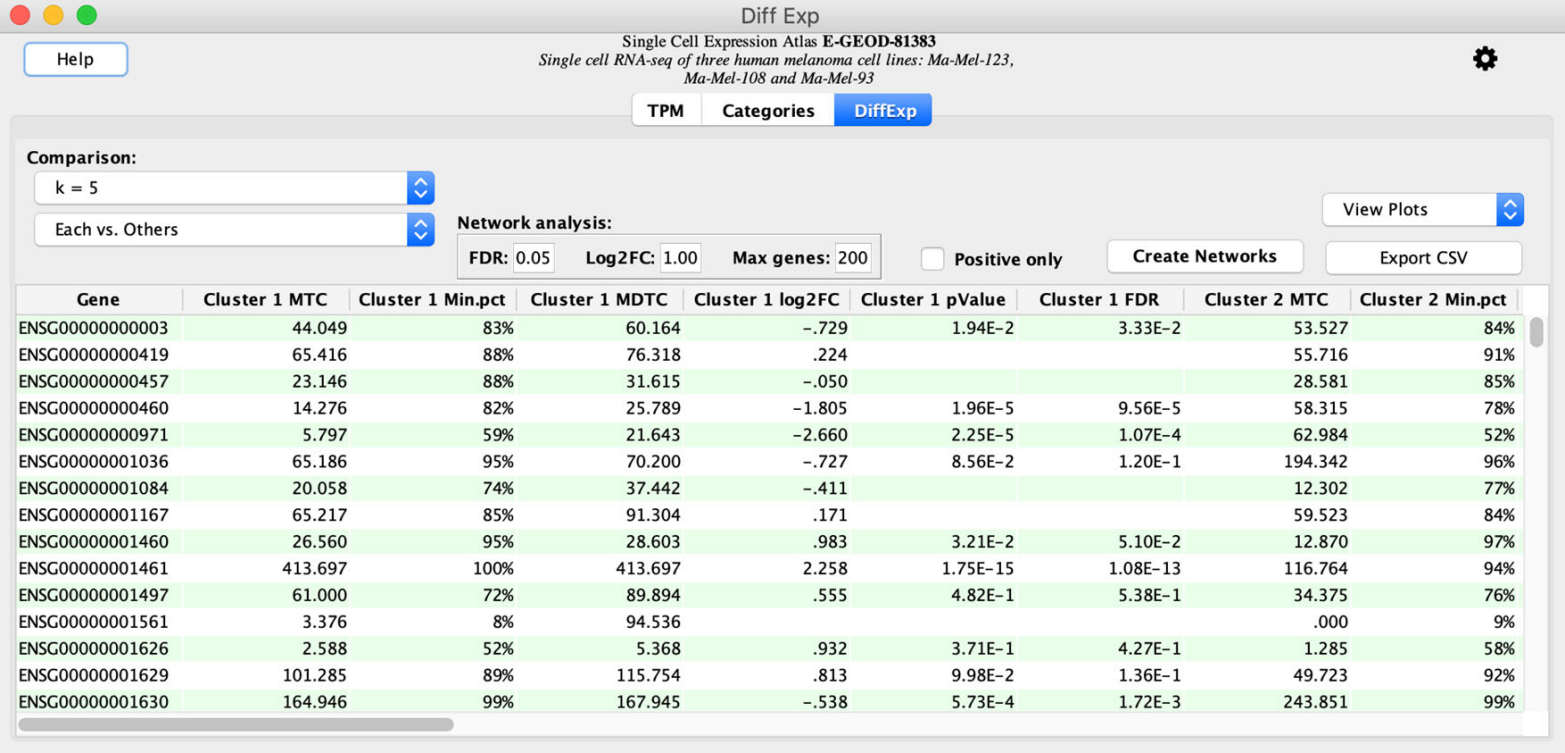
The DiffExp tab allows selection of cutoffs for genes to be used for network analysis and contains options for generating gene by cell heatmaps and violin plots showing gene expression distributions across cell annotation categories.

**Figure 3.**
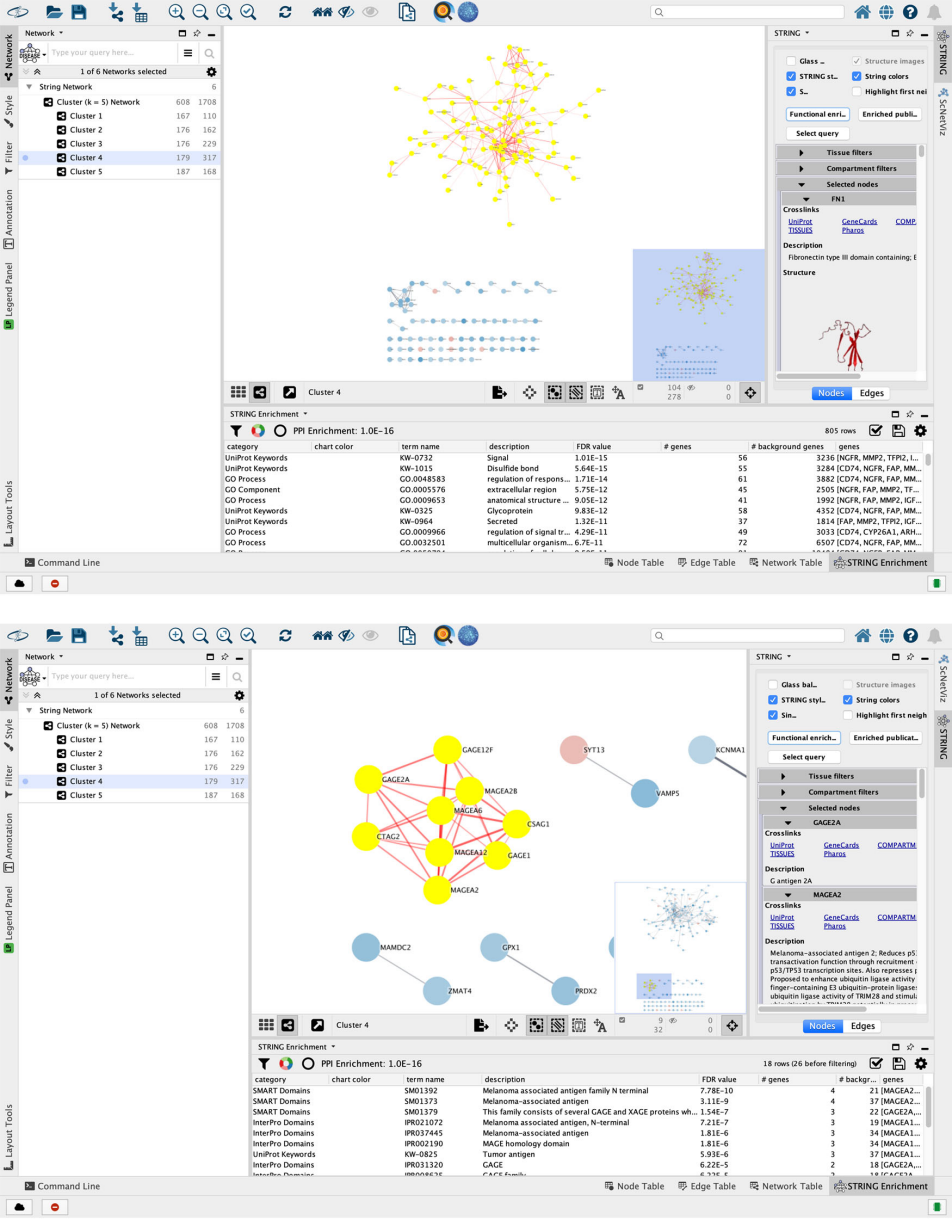
The networks from the STRING database are displayed in the main Cytoscape window. The illustration shows the interaction network for genes specific to the group labeled ”Cluster 4”. Users can filter the complete network using the network analysis tools in Cytoscape. (a) A selection of the largest connected component, which is highlighted with yellow nodes and red edges. (b) Other sub-networks might also be of interest. The second-largest connected component of Cluster 4 shows enrichment of melanoma-associated terms, which is consistent with the fact that this experiment is on melanoma cell lines.

1. Click the 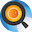 icon in the Cytoscape toolbar. This opens the Single Cell Experiment Atlas browser. For illustration, let us select the experiment with Accession E-GEOD-81383, which contains data on three human melanoma cell lines. ^28^ This is a dataset of 226 cells from short-term cultures of cell lines derived from subcutaneous metastases. To find this experiment, click the column header labelled **Accession** and search for E-GEOD-81383 in the resulting table sorted by accession numbers. Select the row with the accession number E-GEOD-81383 by clicking on it. Note that all the steps of this workflow except for functional enrichment analysis can be executed by selecting an experiment and clicking the button labeled **Create Networks** in the Browse interface. Default parameters for the differential expression analysis and network creation steps can be altered by clicking the gear icon.
2. Click the button labeled **View Data**. This loads the data and opens an experiment table with three tabs.
  - The **Categories** tab lists the annotation categories of cells available in the experiment, which represent grouping of cells by cluster labels for this dataset but may represent grouping by cell types or other criteria. Throughout this manuscript, *categories* refers to cell annotation categories. There are two options listed under **Available categories**. ”Cluster” is the unsupervised grouping of cells computed by the Single Cell Experiment Atlas using the Louvain algorithm ^29^ for a range of resolution parameters. Each value of the resolution parameter yields a different number of clusters, which is called the **K** value (second column of the displayed table as in Figure 1). In keeping with EBI’s practice, by default, *scNetViz* selects the **K** value corresponding to the resolution of 1 for differential analysis and visualization (indicated with a TRUE value corresponding to the selected **K** in the column titled **sel.K**; see Figure 1). ^30^ The other columns show single cell identifiers and their cluster memberships. To get the table of differentially expressed genes press the button labeled **Calculate Diff Exp**. This initiates a comparison of each cluster for K=5 with other clusters.
  - The results are displayed in the **DiffExp** tab. Click the **Create Networks** button. This fetches protein-protein networks for the proteins encoded by genes that satisfy the **Network Analysis** cutoffs based on **FDR** (maximum false discovery rate), **Log2FC** (minimum absolute value of log base 2 of the fold change in expression), and **Max genes** (maximum number of selected proteins; see Figure 2).
3. The networks are listed in the Control Panel (left side) and individually displayed in the main Cytoscape Desktop window ( Figure 3). There are six networks, one for each of the five clusters and one for all the clusters collectively. Select the part of the network that is of interest by holding down the left mouse button on the canvas background and dragging the mouse while holding down the Shift or Ctrl key (Command on Mac).
4. Along the right edge of the Cytoscape window in the Results Panel, click on the **STRING** tab (see Figure 3). Click on the button labeled **Functional Enrichment** to retrieve the enriched pathways and ontology terms in the selected part of the network. The results are displayed in the **STRING Enrichment** tab in the Node Table at the bottom. They can be filtered for, say, pathway terms using the Filter enrichment table option located on the top left of the tab and represented with a black funnel icon.

### Use Case #2: Loading your own scRNA-Seq data from local files for scNetViz analysis

In this example, we will import normalized scRNA-seq data and cluster assignments from local files, visualize all cells in a UMAP plot, perform differential expression analysis based on one of the provided categories, visualize as a combined gene expression heat map, and generate networks for the most significantly differentially expressed genes from each group within the chosen category.

**Figure 4.**
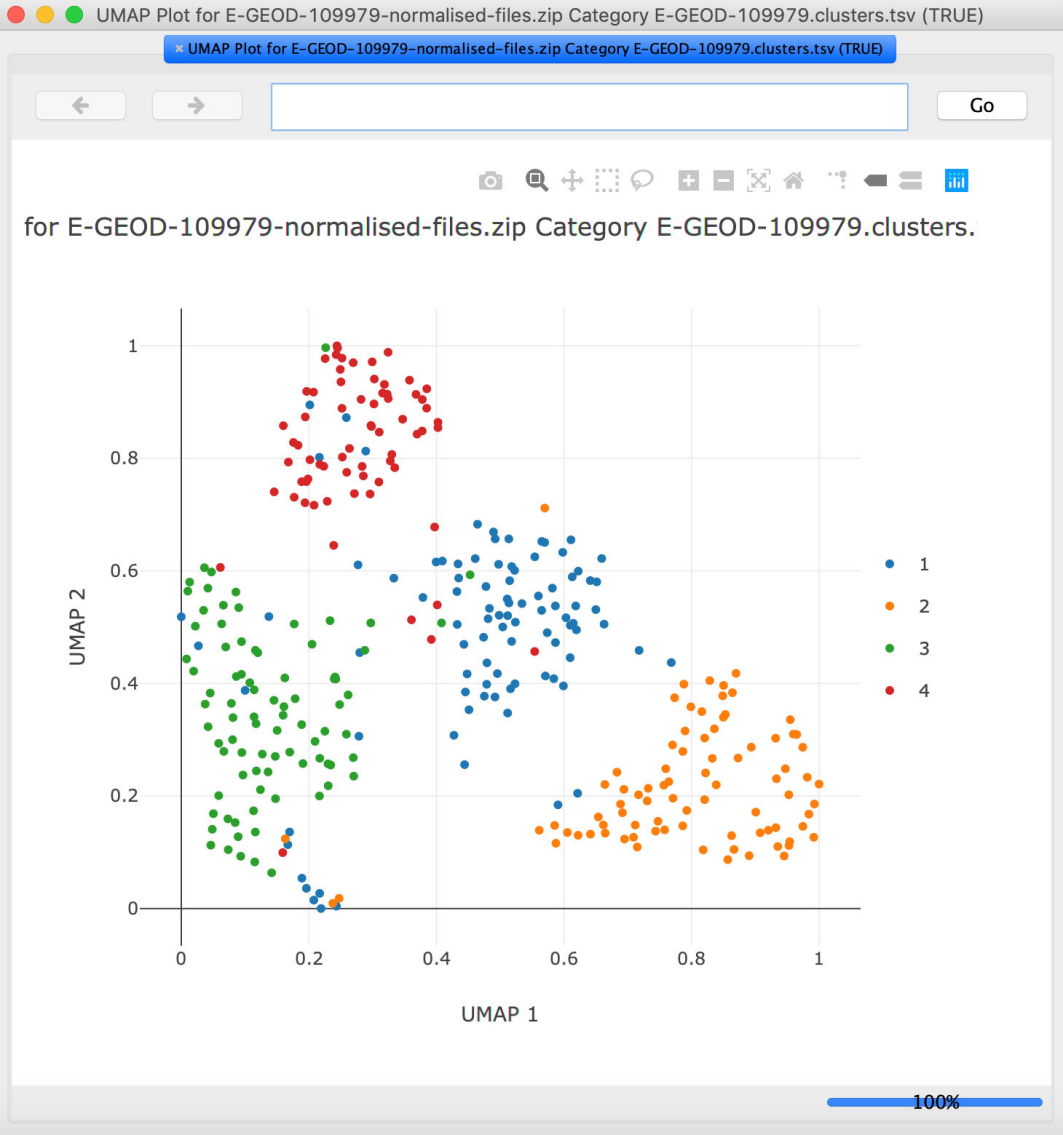
The UMAP plot is displayed in a pop-up window and can be explored interactively. Each dot is a cell, and coloring is by cluster number. Note that the illustration shows results obtained from analysis with the default settings.

**Figure 5.**
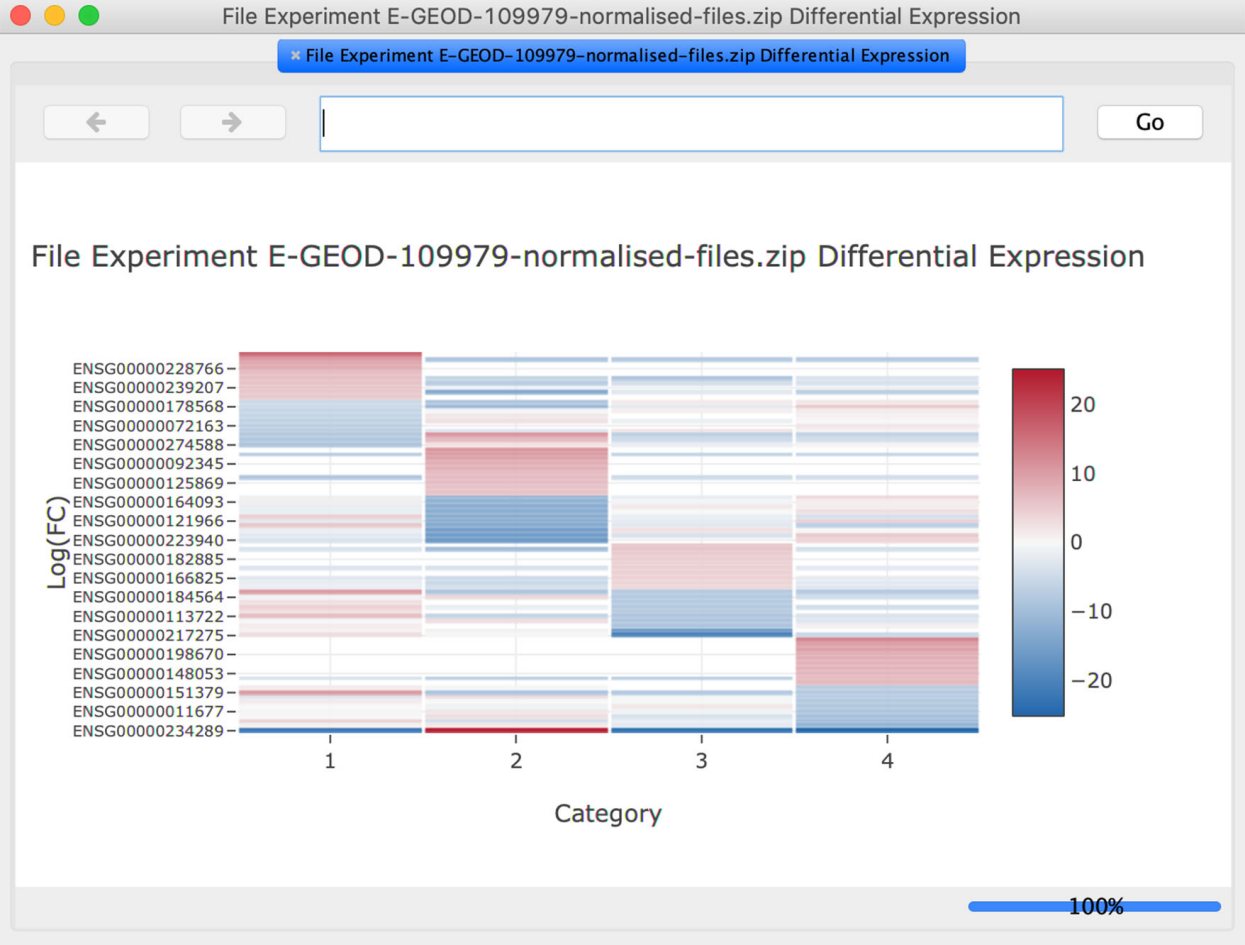
The heatmap for the top differentially expressed genes is displayed in a pop-up window and can be explored interactively. Note that the illustration shows results obtained from analysis with the default settings.

1. Choose **Apps → scNetViz → Load Experiment → Import from file** from the menu, then browse to locate and open a zip, tar.gz, tgz, or gzip file of the three MatrixMarket files (.mtx, .mtx_cols, .mtx_rows) comprising a normalized scRNA-seq quantification dataset. These files can be obtained by normalizing standard outputs from an scRNA-seq processing software such as CellRanger. ^31^ For illustration, we use an experiment on 329 cells at different time points of differentiation starting from human embryonic stem cells (download the normalized counts files and clustering results for accession ID E-GEOD-109979 from the Single Cell Expression Atlas ^32^). In the dialog, select the species, which is *Homo sapiens* for this data.
2. From the window with the experiment table, load the downloaded clustering results as category data using the **Import from file** option in the menu under **Add Category**. Alternatively, select the option for **Louvain clustering** or **Leiden clustering** from this menu to perform unsupervised clustering of the cells.
3. From the **Categories** tab, select the row with the grouping of cells that should be highlighted on the UMAP plot. Select the **UMAP** option from the **New Cell Plot** menu. If required, change the options and press **OK** to view the UMAP plot ( Figure 4).
4. Switch to the **Categories** tab on the experiment table window. Select a category by clicking its row and press **Calculate Diff Exp**. This switches the display to the **Diff Exp** tab.
5. Select the **Heatmap** option from the **View Plots** menu on the **Diff Exp** tab. This generates a gene expression heatmap for genes that meet the filtering criteria ( Figure 5).
6. Click the **Create Networks** button on the **Diff Exp** tab to fetch the protein functional interaction networks for the top genes.

## Conclusion

*scNetViz* is a Cytoscape app for identifying differentially expressed genes characteristic of cell clusters and displaying networks of the corresponding proteins for further analysis. This workflow aids interpretation of cell clusters from scRNA-seq and related single-cell resolution data. It provides several ways of plotting cells and gene expression data, e.g., heatmap, violin, t-SNE, and UMAP plots. We describe two use cases demonstrating its utility for integrating network information with local or public single-cell RNA sequencing data. Users can set the analysis parameters, save settings, analyze their own data or load data from public atlases, and interactively select gene sets or cell clusters for network analysis. *scNetViz* is highly portable to diverse computational systems via its GUI or CyREST services. It only has Cytoscape and its *stringApp*, *cyBrowser*, *cyPlot* and *enhancedGraphics* apps as dependencies. Compared with other single cell analysis tools it provides a user-friendly interface and no coding expertise is needed for its use.

Currently, *scNetViz* does not have options to normalize or batch-correct raw counts. To analyze their own data, users must input normalized and batch corrected data. The methods for normalization, batch correction, clustering, and differential expression analysis are under active development and benchmarking. As the standard workflow of analysis evolves, we will continue to integrate newer methods in *scNetViz.* For differential analysis, *scNetViz* only supports comparing a group of cells within a category with the set of cells in all other groups in that category combined (e.g. to identify cell-type-specific genes, or gene expression markers). In the future, we will support more general comparisons, including comparison of any two groups of cells provided or selected by the user. Future development will also enable excluding pseudogenes or other gene sets from the analysis. File formats are also evolving. For instance, the Loom format is becoming more popular. This format is not supported by *scNetViz* currently. As new standards are adopted for single-cell data, we will add support for them.

## Software availability

• Software available from the Cytoscape App Store: https://apps.cytoscape.org/apps/scnetviz
• Software code available from GitHub: https://github.com/RBVI/scNetViz
• Archived source code at the time of publication: https://doi.org/10.5281/zenodo.4641480 ^33^
• License: Apache License, Version 2.0
• Online manual: https://www.cgl.ucsf.edu/cytoscape/scNetViz/index.shtml
• *scNetViz* Rmd and Python notebooks: http://automation.cytoscape.org

## Data availability

The source data used for the use cases are available from Single Cell Expression Atlas.

• Use Case #1: https://www.ebi.ac.uk/gxa/sc/experiments/E-GEOD-81383/downloads
• Use Case #2: https://www.ebi.ac.uk/gxa/sc/experiments/E-GEOD-109979/downloads
