# Supplementary material for: scNetViz: from single cells to networks using Cytoscape
Source: F1000Res. 2021 Jun 7;10:ISCB Comm J-448. [Version 1] doi: 10.12688/f1000research.52460.1 (PMC8593621; doi:10.12688/f1000research.52460.1)
Supplement: Peer review history

# Peer review: Reviewer response for version 1

**DOI:** 10.5256/f1000research.55741.r98565

**Published:** 2021-11-15

**Authors:** Kenji Kamimoto

**Affiliations:** 1 Department of Developmental Biology, Washington University School of Medicine in St. Louis, St. Louis, MO, USA

The authors have developed scNetViz, a Cytoscape-based software for constructing and analyzing cluster-specific protein-protein interaction networks from scRNA-seq data.

 scNetViz selects genes differentially expressed in each cluster based on scRNA-seq data and constructs a cluster-specific network by filtering the universal protein-protein interaction network registered in the STRING database. The system includes all necessary steps, including data search, download, clustering, network filtering, and visualization. All these operations can be performed with the GUI, enabling easy and intuitive network analysis.

 The installation of scNetViz by Cytoscape's App Manager is simple, and I could install scNetViz without any trouble. The use cases presented in the paper help the reader to understand the typical usage of scNetViz, and they are described clearly. The analysis with a Single Cell Expression Atlas could be conducted without any problem.

 scNetViz creates a network by filtering the STRING protein-protein interaction network database. Although this concept is described in the introduction and later sections, it is not clear from the title and abstract of the paper (version 1). Thus, the title and abstract might make the wrong impression that scNetViz can infer a *de novo* gene regulatory network from scRNA-seq data rather than filtering the pre-existing network. Therefore, to clarify the concept of the scNetViz paper, I would like to recommend adding descriptions of the following points in the abstract or title.

1. scNetViz is a network filtering analysis, not a *de novo *GRN inference algorithm.
2. scNetViz uses protein-protein interaction network analysis, and it is not another GRN analysis such as co-expression or transcriptional gene regulatory network.

Are the conclusions about the tool and its performance adequately supported by the findings presented in the article?

Yes

Is the rationale for developing the new software tool clearly explained?

Yes

Is the description of the software tool technically sound?

Yes

Are sufficient details of the code, methods and analysis (if applicable) provided to allow replication of the software development and its use by others?

Yes

Is sufficient information provided to allow interpretation of the expected output datasets and any results generated using the tool?

Yes

Reviewer Expertise:

Computational Biology

I confirm that I have read this submission and believe that I have an appropriate level of expertise to confirm that it is of an acceptable scientific standard.

# Peer review: Reviewer response for version 1

**DOI:** 10.5256/f1000research.55741.r86919

**Published:** 2021-07-26

**Authors:** Luca Pinello

**Affiliations:** 1 Molecular Pathology Unit, Center for Cancer Research, Massachusetts General Hospital, Department of Pathology, Harvard Medical School, Boston, MA, USA

Choudhary *et al.,* present scNetViz a useful Cytoscape app to perform network analysis on single-cell RNA-seq data at cluster level.

 The manuscript is well written and clear. The software is well documented with clear examples, use cases and nice illustrative figures.

 Particularly helpful is the possibility to load datasets from several public repositories (EMBL-EBI and Human Cell Atals) with simple dedicated functions.

 Some points to consider to improve the manuscript and software:

1. The documentation regarding the installation of the software. For example on OSX it is very tricky to install and use it since Java applications that are not signed are not allowed and a warning message is displayed. This may discourage several users, so it could be helpful to have step by step and explicit instructions that cover this case and also that assume a user doesn't have yet Cytoscape installed. On Windows 10 things are much easier and I was able to install it by using the Apps-> App Manager... function.
2. The search text are on the window "Single cell Expression Atlas Browser" doesn't work properly and I was not able to select E-GEOD-8138 with this function (it doesn't scroll automatically to the correct line).
3. I was able to reproduce the analysis for E-GEOD-81383, however the tool failed to create a network for E-CURD-3 when clicking "Create Networks " (the first dataset presented on the provided list). If not all the datasets are supported, it would be helpful showing to the users only the ones where it is possible to create networks and explaining why it is not possible to build networks for some datasets.

Are the conclusions about the tool and its performance adequately supported by the findings presented in the article?

Yes

Is the rationale for developing the new software tool clearly explained?

Yes

Is the description of the software tool technically sound?

Yes

Are sufficient details of the code, methods and analysis (if applicable) provided to allow replication of the software development and its use by others?

Partly

Is sufficient information provided to allow interpretation of the expected output datasets and any results generated using the tool?

Yes

Reviewer Expertise:

Computational Biology

I confirm that I have read this submission and believe that I have an appropriate level of expertise to confirm that it is of an acceptable scientific standard.
